# Gene analysis for longitudinal family data using random-effects models

**DOI:** 10.1186/1753-6561-8-S1-S88

**Published:** 2014-06-17

**Authors:** Jeanine J Houwing-Duistermaat, Quinta Helmer, Bruna Balliu, Erik van den Akker, Roula Tsonaka, Hae-Won Uh

**Affiliations:** 1 Department of Medical Statistics and Bioinformatics, Leiden University Medical Center, PO Box 9600, 2300 RC, Leiden, The Netherlands; 2 Department of Molecular Epidemiology, Leiden University Medical Center, PO Box 9600, 2300 RC, Leiden, The Netherlands; 3 The Delft Bioinformatics Lab, Delft University of Technology, PO Box 5031, 2600 GA, Delft, The Netherlands

## Abstract

We have extended our recently developed 2-step approach for gene-based analysis to the family design and to the analysis of rare variants. The goal of this approach is to study the joint effect of multiple single-nucleotide polymorphisms that belong to a gene. First, the information in a gene is summarized by 2 variables, namely the empirical Bayes estimate capturing common variation and the number of rare variants. By using random effects for the common variants, our approach acknowledges the within-gene correlations. In the second step, the 2 summaries were included as covariates in linear mixed models. To test the null hypothesis of no association, a multivariate Wald test was applied. We analyzed the simulated data sets to assess the performance of the method. Then we applied the method to the real data set and identified a significant association between *FRMD4B *and diastolic blood pressure (*p*-value = 8.3 × 10^-12^).

## Background

Testing for the joint effect of single-nucleotide polymorphisms (SNPs) located in a gene is a popular alternative to single-marker tests. Single SNP methods are underpowered because single SNPs have typically small effect sizes (common variants) or small minor allele frequencies (MAFs) (rare variants). In contrast, approaches that model the combined effect of multiple SNPs will be more powerful. Recently, we have proposed a method consisting of 2 steps [1,2]: (a) the dimensionality of the genetic data is reduced, and gene-specific summaries are produced, and (b) these summaries are introduced as covariates in the model for the phenotype. To model the correlation among SNPs within a gene, we use a generalized linear mixed model for the SNPs. A gene-level random effect captures the correlation within each gene. The empirical Bayes estimates of the random effects per subject and gene are used as summary measures of the SNPs data and are included in the phenotype model to test for association.

For the Genetic Analysis Workshop 17 (GAW17), we studied the performance of this approach for the sequence data on the families [2]. The conclusion was that common variants were well represented by this gene summary, but variation due to rare variants was not well captured. For rare variants several collapsing methods have been developed (see [3] for an overview).

Using the GAW18 data, we extend our 2-step method to the combined familial and longitudinal setting. Next to the empirical Bayes estimates, we will also consider the total number of rare variants (MAF < 0.05) to summarize the information on rare SNPs within a gene. Using the 200 simulated data sets, we assess the power of our approach for genes with functional loci. We compare results obtained by using the original Variant Call Format (VCF) files as well as the files in which the genotypes of relatives having only genome-wide association (GWA) data were imputed. Gene summaries will be computed based on all variants and based on only functional variants. For the simulated data sets, we assume that both gene effects are constant over time. Finally, we present the results of analyzing the true data set for associations with diastolic blood pressure (DBP). In this data set, we have identified one significant result when using the VCF files, assuming no interactions between gene summaries and time, and 2 borderline significant associated genes when we included an interaction between time and gene effects to allow gene effects to change over time. Because of the computational burden, we consider only chromosome 3.

## Methods

### Study sample

We considered data for 959 individuals from 20 families. Of these, 464 are directly sequenced individuals; for their family members, imputed WGS data based on the existing GWA framework were available. In this article, we restrict ourselves to genotypic data from chromosome 3. For each individual, we have information on age at examination and current tobacco smoking (yes = 1; no = 0) for up to 4 time points for real data and for up to 3 points for the simulated data. We analyzed the quantitative trait DBP. There are 1274 genes on chromosome 3. From these, 927 and 929 genes contain at least one rare variant for the imputed files and one for the VCF files. When restricting the analysis to functional variants, these numbers are 46 and 43, respectively. To extract genes, we used the R packages GenomicFeatures and RSQLite from Bioconductor. We used the tool ANNOVAR [4] with the UCSC Known Genes database [5] to select the exonic SNPs and their predicted effect on the protein. If this effect is known and not synonymous (e.g., nonsynonymous, or stop codon introducing or removal), the SNP was considered functional. See Almasy et al [6] for a more extended description of the data set.

### Model specification

Let *y_ijt _*be the outcome variable for individual *j *from family *i *at time point *t*. For a specific gene *g*, let *w_ijgs _*be the genotype at SNP *s *(*s = *1,*..., S*). The genotype *w*_*ijgs*_ is coded 0, 1, or 2. For individual *j *of family *i*, let *x_ijt _*be vectors with covariate values for the phenotypes (age and smoking status).

### Gene summaries

We assume that Hardy Weinberg equilibrium holds. We consider a random gene effect to model the correlation among SNPs within a gene. Let *b_jg _*be the random gene effect of gene *g *for subject *j*. Given this random effect, *w*_*ijgs*_ , the number of minor variants for SNP *s *in gene *g *is assumed to follow a binomial distribution with *n *= 2 trails and probability π_*ijgs.*_ The probability π_*ijgs*_ is modeled as follows:

(1) $$\text{log}\frac{\pi_{ijgs}}{1-\pi_{ijgs}}=\alpha +b_{jg},$$

where *b_jg _*follows a normal distribution with zero mean and variances σ^2^. For each individual and each gene, the empirical Bayes estimate is given by $e{\hat{b}}_{ijg}={\hat{b}}_{jg}$. Intuitively, the value of the empirical Bayes estimate will increase with the number of variants a subject carries. These models are fitted using the package lme4 in R. Because rare variants are not well captured by the empirical Bayes estimates, we also consider the total number of rare variants *s_ijg _*(MAF <0.05) as a second summary measure of the genetic information per subject.

### Phenotype model

Both the empirical Bayes estimates and the number of rare variants in a specific gene can be plugged into the models for the phenotypes to test for gene effects. To model the longitudinal quantitative trait DBP, we use the following linear mixed model for each gene *g*:

(2) $$Y_{ijt}=\mu +\beta x_{ijt}+\gamma_{1}eb_{ijg}+\gamma_{2}s_{ijg}+u_{ij}+e_{ijt},$$

with *s*_*ijg*_ the number of rare variants within gene *g, u*_*ij*_ a normally distributed random family effect, and *e_ijt _*a normally distributed residual with a *T *times *T *covariance matrix to model the correlation between repeated measurements within a person (unstructured covariance error term). Here *T *is the number of time points. The variance of the family effect *u*_*ij *_is equal to variance τ^*2*^ and the correlation of *u_i_=(u_i1_....u_ini _*) within a family of size *n_i _*is assumed to be equal to 2 times the kinship coefficient between the relatives (polygenic). Estimates of all model parameters including the *T(T+1)/2 *parameters of the unstructured covariance matrix were obtained by maximizing the likelihood function using the optim function in R [7]. Based on Model (2) we can test the null hypothesis of no gene effect, which is equivalent to testing the null hypothesis H_0_: γ_1_=γ_2 _= 0. We used a multivariate Wald statistic with 2 degrees of freedom. In addition, to model time-dependent gene effects we added interaction terms between the gene effects (*eb *and *s*) and the time variable. The corresponding multivariate Wald tests for association of a gene (cross-sectional and over time) will have 2*T *degrees of freedom; *p*-values smaller than 6 × 10^-5 ^were considered to be statistically significant (Bonferroni correction) while *p*-values smaller than 10^-4 ^were considered to be borderline significant.

## Results

### Power

The maximum power was achieved for *MAP4 *gene using the imputed files and restricting the analysis to functional variants (96.5% power for the 2 degrees of freedom test). For all variants based on imputed files the power for this gene was only 36.5%. For functional variants based on the VCF files the power was 72.5%. Indeed, the total percentage of variance explained by the loci for *MAP4 *was largest among the genes at chromosome 3, namely 0.0648 of all associated genes.

To show the performance of our methods for various genetic effects, we also provide each gene with functional loci (Supplemental Table 1 of GAW18 answers) the percentage of data sets with a *p*-value smaller than 0.05 in Table 1. The results for the univariate Wald tests and the multivariate Wald tests are given. In addition to *MAP4 *gene*, RYBP, ZBTB38*, and *GPR160 *had in more than 10% of the data sets a *p*-value smaller than 5% for the multivariate test. For *RYBP *and *GPR160*, the sum of rare variants showed a better performance than the empirical Bayes estimate. Indeed, *RYBP *has 2 functional loci with MAFs less than 0.05, and *GPR160 *has one functional locus with a MAF less than 0.05. For *ZBTB238 *and *MAP4*, the empirical Bayes gene summary performed better. Gene *ZBTB238 *has 2 functional loci with MAFs less than 0.05. Because the effects are opposite, the sum score has no power. Gene *MAP4 *has 12 functional loci with MAFs less than 0.05. Seven of these loci carriers of the rare variant had a smaller DBP, and 5 loci carriers of the rare variant had a higher DBP than noncarriers.

**Table 1:** Power based on analysis of genes at chromosome 3 in simulated datasets.

Gene	Number of variants	% Variance of largest functional variant	Power of *eb*^1^	Power of *s*^2^	Power of combined^3^
*PDCD6I*	466	0.00040	0.0	7.0	2.5
*DNASE1L3*	115	0.00014	2.0	4.5	1.5
*PTPLB*	493	0.00002	3.5	8.5	5.5
*PAK2*	409	0.00005	3.5	0.6	4.0
*FBLN2*	687	0.00008	0.5	3.0	0.5
*FLNB*	956	0.00085	11.0	5.0	7.0
*VPS8*	1042	0.00008	3.0	12.0	5.0
*RYBP*	347	0.00041	6.0	21.0	15.5
*ZBTB38*	590	0.00031	51.0	4.0	34.5
*GPR160 2*	44	0.00020	3.0	19.5	12.0
*SERP1*	18	0.00002	0.0	5.0	1.5
*SUMF1*	747	0.00010	2.0	1.0	1.0
*NMNAT3*	559	0.00011	5.0	5.5	6.5
*ARF4*	161	0.00004	1.5	3.5	2.0
*MAP4*	894	0.01222	99.0	30.5	97.0
*MLH1*	310	0.00007	2.5	2.0	1.5
*ARHGEF3*	2223	0.00007	2.0	5.0	3.0
*PPP2R3A*	1081	0.00025	0.5	1.0	1.0
*MUC13*	203	0.00007	5.0	0.5	3.0
*RAD18*	693	0.00003	3.0	3.5	3.5
*SEMA3F*	134	0.00004	12.0	7.5	6.5
*BTD*	291	0.00011	1.0	2.5	1.0
*ABTB1*	48	0.00053	3.5	1.1	6.5
*B4GALT4*	217	0.00004	2.0	4.5	3.0

The values represent the percentages of significant results at 5% level. Imputed data sets were used.

1 Empirical Bayes estimate H_0_:γ_1 _= 0.

2 Number of rare variants H_0_:γ_2 _= 0.

3 H_0_:γ_1 _= 0 and γ_2 _= 0.

Overall, the percentage of genes with a significant result at the 5% level appeared to be 7.1%.

### Analysis of real data set

For the real data set, we did not find any significant results when we used the imputed files. The smallest *p*-value was 0.002 for gene *COX17*. When using the VCF files, we identified a significant association between *FRMD4B *and DBP (*p-*value = 8.3 × 10^-12^). The total number of variants in this gene was 2348; 1388 SNPs had MAFs smaller than 5%. The *p*-value for the empirical Bayes estimate was 5.3 × 10^-6 ^and for the number of rare variants, 0.057. When using the imputed files, the *p*-value for this gene was only 0.32.

**Figure 1:**
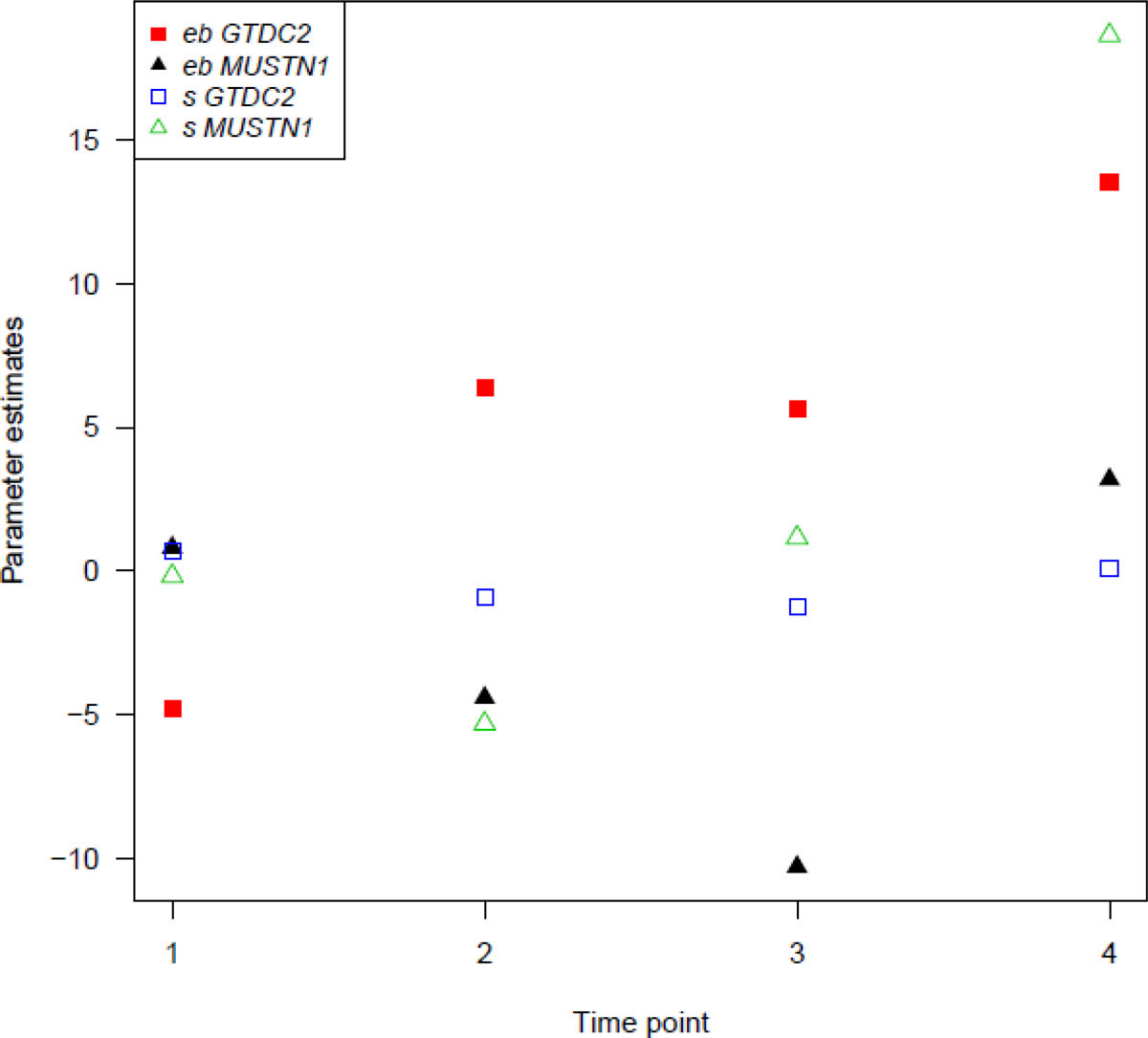
**Estimates of the model parameters γ_1 _and γ_2._**For *MUSTN1 *and *GTDC2*, estimates of the model parameters γ_1 _and γ_2 _for empirical Bayes estimates *eb *and number of rare variants *s *for the 4 time points.

When we included an interaction term between the gene summaries and time, we identified 2 more genes showing borderline significance when using the VCF files. The genes are *MUSTN1 *and *GTDC2 *with *p*-values of 7.5 × 10^-5 ^and 9.9 × 10^-5^, respectively. For both genes, the association between the rare variants and DBP appears to be largest (smallest *p*-values). For *MUSTN1*, the effect is most pronounced for the first time points; for *GTDC2*, the association is most significant for the last time points. The effect sizes are depicted in Figure 1. For *GTDC2*, the parameter estimates for the *eb *gene summary increase over time.

## Discussion

We used a 2-step procedure in which, first, the information in a gene is summarized by 2 variables, namely the empirical Bayes estimate capturing common variation and the number of rare variants. In the second step, these summaries are included as covariates in linear mixed models. Interactions such as time-gene summary can be included. The results of the analysis of real data showed that time-gene interactions may identify other genes. Another advantage is that this method can deal with missing data. An alternative method is generalized estimating equations (GEEs). However, existing R-modules do not allow for flexible correlation structures, resulting in a conservative approach when the working correlation does not agree with the true correlation structure. Moreover, whereas the GEE method assumes complete missing at random, mixed models are valid under the missing at random assumption.

Using the linear mixed model without an interaction effect between time and gene summaries for the real data set, we identified one significant gene when using the VCF file, *FRMD4B*. The association of this gene appears to be biologically sound. It has been found to be associated with heart failure [8]. Using the real data set, we did not find any association using the imputed data sets. Probably, the followed imputation procedure provided noise. In addition, 2 genes showed borderline significance when allowing the effect of the gene summaries to change over time.

With regard to the power of the method, the power was good only for *MAP4*. Restriction of the analysis to functional variants improved the performance considerably. This can be explained by the fact that in the simulation model, only the "functional" loci are associated. Note that for the real data, significant results were obtained only when all SNPs were used. This shows that restricting to functional variants may result in false negatives.

The empirical Bayes summary appeared to perform well. The sum of rare variants, however, does not perform well when variants have opposite effects. In addition, the sum score does not assign more weights to a variant that segregates within the family compared with a rare variant that occurs in several families. To include this information in testing for association of rare variants will require future research.

## Conclusions

The 2-step approach is a flexible method for performing a gene-based analysis: it can be used for any design and can model time-dependent effects in longitudinal designs. For this relatively small sample size, this approach was able to detect genes that explain 0.0648% of the variance (power of 97%). With regard to the real data set, the association between gene *FRMD4B *and DBP was significant (*p *= 8.3 × 10^-12^).

## Competing interests

The authors declare that they have no competing interests.

## Authors' contributions

JJH-D developed the method, interpreted the results, and wrote the manuscript. QH analyzed the data and interpreted the results. BB participated in analysis of the data and interpretation of the results. EA developed the tools for efficient analysis of sequencing data sets and read the paper. HWU developed the statistical method, interpreted the results, and participated in writing the paper. RT developed the statistical methods and participated in writing the paper.

## References

[B1] Tsonaka R van der Helm-vander Mill A Houwing-Duistermaat JJ Association tests for the effect of genetic pathways on longitudinal outcomes Stat Med 2012 31 1190 1202 21997511 10.1002/sim.4370

[B2] Houwing-Duistermaat JJ Uh HW Tsonaka R Pathway analysis for family data using nested random-effects models BMC Proc 2011 5 suppl 9 S22 10.1186/1753-6561-5-S9-S22 22373228 PMC3287857

[B3] Ladouceur M Dastani Z Aulchenko YS Greenwood CM Richards JB The empirical power of rare variant association methods: results from Sanger sequencing in 1,998 individuals PLoS Genet 2012 8 e1002496 10.1371/journal.pgen.1002496 22319458 PMC3271058

[B4] Wang K Li M Hakonarson H ANNOVAR: functional annotation of genetic variants from next-generation sequencing data Nucleic Acids Res 2010 38 e164 10.1093/nar/gkq603 20601685 PMC2938201

[B5] UCSC Known Genes database http://hgdownload.cse.ucsc.edu

[B6] Almasy L Dyer TD Peralta JM Jun G Fuchsberger C Almeida MA Kent JW Jr Fowler S Duggirala R Blangero J Data for Genetic Analysis Workshop 18: human whole genome sequence, blood pressure, and simulated phenotypes in extended pedigrees BMC Proc 2014 8 suppl 2 S2 10.1186/1753-6561-8-S1-S2 PMC4145406 25519314

[B7] R program http://www.r-project.org

[B8] Cappola TP Li M He J Ky B Gilmore J Qu L Keating B Reilly M Kim CE Glessner J Common variants in HSPB7 and FRMD4B associated with advanced heart failure Circ Cardiovasc Genet 2010 3 147 154 20124441 10.1161/CIRCGENETICS.109.898395 PMC2957840

